# Linking Self-Esteem to Problematic Mobile Phone Use: A Moderated Mediation Model of Fear of Missing Out and Ruminative Subtypes

**DOI:** 10.3390/bs14080683

**Published:** 2024-08-06

**Authors:** Xiujuan Yang, Qingqi Liu, Lingfeng Gao, Guojun Wang, Tiebang Liu

**Affiliations:** 1 State Key Laboratory of Chemical Oncogenomics, Guangdong Provincial Key Laboratory of Chemical Genomics, Peking University Shenzhen Graduate School, Shenzhen 518055, China; yxj@skh.net; 2 Shenzhen Mental Health Center, Shenzhen Kangning Hospital, Shenzhen 518118, China; wangguojun@skh.net; 3 Department of Psychology, Faculty of Arts and Sciences, Beijing Normal University, Zhuhai 519087, China; liuqingqi@bnu.edu.cn; 4 Research Center of Adolescent Psychology and Behavior, School of Education, Guangzhou University, Guangzhou 510006, China; 5 Institute of Psychological and Brain Sciences, Zhejiang Normal University, Jinhua 321000, China; glfpsy@zjnu.edu.cn

**Keywords:** self-esteem, problematic mobile phone use (PMPU), fear of missing out (FoMO), rumination, brooding, reflection

## Abstract

Low self-esteem has been identified as a risk factor for problematic mobile phone use (PMPU). However, the magnitude of self-esteem’s effect on PMPU varied across different studies. Drawing on the Interaction of Person-Affect-Cognition-Execution model and the response styles theory, this study developed a moderated mediation model to investigate the mediating role of fear of missing out (FoMO) and the moderating roles of ruminative subtypes (i.e., brooding and reflection) in the relationship between self-esteem and PMPU. We conducted a cross-sectional survey among 806 undergraduate students (*M*_age_ = 19.35 years, *SD* = 1.18) using the convenience sampling method. Results showed that self-esteem was negatively associated with PMPU. Mediation analysis revealed that the association between self-esteem and PMPU was mediated by FoMO. Furthermore, moderated mediation analyses revealed that the mediating effect of FoMO was moderated by both brooding and reflection, such that the indirect effect became stronger for individuals with higher levels of brooding/reflection. These findings add to previous research by shedding light on how (i.e., mediation) and under what conditions (i.e., moderation) self-esteem is associated with PMPU and have implications for early prevention and intervention of individual PMPU.

## 1. Introduction

The dramatic increase in digital technology use in modern society has led to growing concerns about people’s problematic use of digital technologies (e.g., mobile phones) [1,2,3,4]. According to the fifth edition of the Diagnostic and Statistical Manual of Mental Disorders [5], internet gaming disorder was listed as a potential behavioral addiction that exerts profound impacts on individual mental health [4,6]. Mobile phones have become an indispensable medium in modern society, integrating a wide range of advanced applications. For instance, people can socialize on social networks (e.g., WeChat, Facebook, and Instagram), play mobile games, watch videos for entertainment, search for information online, and engage in online shopping, learning, and cross-cultural communication. Due to their convenience, customization, and multifunctionality, mobile phones may exhibit some characteristics akin to addictive behaviors, particularly concerning the problematic use of mobile phones.

Problematic mobile phone use (PMPU), also known as mobile phone addiction or mobile phone dependency, refers to individuals’ inability to regulate their use of mobile phones, which eventually has a negative impact on daily life [7,8]. The definition of PMPU can be understood from two core elements: (a) a compulsive pattern of use characterized by maladaptive dependency on mobile phones and a tendency to excessively use them regardless of negative consequences, and (b) the negative consequences arising from excessive mobile phone use including impairment in individual physical, mental, and social functioning [9,10,11]. Some researchers further proposed the diagnostic criteria for negative consequences, including (a) physical and psychological harm, (b) using mobile phones in hazardous situations, (c) negative impacts on social relationships and school or work performance, and (d) subjective distress [11]. Although there have been controversies regarding whether PMPU can be defined as a behavioral addiction [7,12], a growing body of research has validated the psychological and functional impairment due to excessive mobile phone use [9,13,14]. The commonly established symptoms of PMPU include impaired control over mobile phone use characterized by an inability to control the amount of time spent on mobile phones (loss of control), experiencing unpleasant feelings when stopping or reducing mobile phone use (withdrawal), using mobile phones to induce euphoria and/or other mood-regulating experiences (mood modification), and social and family conflicts and loss of interest in other activities (conflict) [11,12,15,16]. Some researchers have further distinguished between different types of PMPU, such as problematic use of mobile social networking, gaming, information acquisition, and short-form video use [17]. The present study will focus on the general problematic use of mobile phones.

The high prevalence of PMPU across cultures and societies has been recognized in prior studies [1,18,19]. A systematic review and meta-analytical study revealed that the overall prevalence rate of problematic smartphone use worldwide was about 26.99% (95% CI = [22.73, 31.73]) [20]. PMPU is generally associated with a wide range of negative outcomes [9,18], including poor sleep quality [18,19], depression and anxiety [21], poor academic outcomes [22], and interpersonal problems [23]. A significant correlation between digital technology addiction and symptoms of psychiatric disorders has also been documented [4,6,24]. Researchers have developed a variety of effective interventions to address PMPU, including exercise and psychological interventions such as cognitive-behavioral therapy (CBT) and educational programs [25,26], school restrictions, peer support, self-awareness, and self-control [27]. However, these intervention studies primarily target individuals identified through self-report or clinical screening as having symptoms of internet addiction, focusing on indicated preventive interventions. This approach may encounter challenges such as high costs and susceptibility to relapses [28]. Building on this foundation, to enhance the effectiveness of these interventions, it is crucial to identify specific psychological processes that influence PMPU and to conduct targeted intervention strategies from a preventive perspective.

Researchers have investigated a host of factors that may relate to PMPU, including gender and age [2,3,9], parental mediation [29], peer relationships [30], sensation seeking [31], rumination, fear of missing out, and depression [32,33]. Self-esteem, as a core personality factor, has also been identified as a crucial factor in the development of PMPU [30,31,34]. However, the effect sizes of self-esteem on PMPU varied across different studies, suggesting that they may be influenced by other variables. This study will extend existing studies by elucidating the pathways through which self-esteem influences PMPU (i.e., mediation) and the conditions under which self-esteem can exert a greater effect on PMPU (i.e., moderation). These findings will provide guidance for formulating preventive intervention strategies to address PMPU.

### 1.1. Self-Esteem and PMPU

The term self-esteem refers to one’s overall evaluation of oneself and the perception of personal worth and competence [35,36]. Self-esteem is a psychological trait that exerts a profound effect on individual psychological adjustment [37] and social relationships [38]. The effects of self-esteem on dysfunctional behaviors have also been demonstrated, including health risk behaviors (e.g., alcohol abuse) [39] and gaming disorders [40]. As digital technology plays an increasingly important role in daily life, the association between self-esteem and problematic digital use (e.g., PMPU) has attracted more scholarly and social attention. Theoretically, according to the excessive reassurance pathway leading to PMPU [7], people with low self-esteem typically hold unfavorable self-views [41] and negative core self-beliefs [34] and have a constant need to maintain relationships and obtain reassurance from others. Mobile phones can provide an idealized platform for people to express positive self-views and seek comfort and relief, potentially raising the likelihood of developing PMPU [7]. Empirical research has documented self-esteem as a vulnerability factor for PMPU; however, the effect sizes of self-esteem on PMPU are inconsistent [42].

Specifically, some studies have provided support for the close relationship between low self-esteem and PMPU [16,43], indicating that people with low self-esteem are more inclined to engage in PMPU. At the same time, a subset of studies have found that in multivariate analyses, the association between self-esteem and PMPU becomes non-significant [44,45]. To address the inconsistent findings regarding self-esteem and PMPU, some researchers have conducted a series of meta-analyses. A meta-analysis of 31 studies observed a negative association between self-esteem and PMPU with a small to medium effect size (Fisher’s Z = −0.25) [34]. Moreover, a meta-analysis focusing on mainland Chinese adolescents revealed a moderate correlation between self-esteem and PMPU (r = −0.25), with this correlation being stronger in secondary school students than college students [46]. Some studies have further examined the mechanisms underlying the relationship between self-esteem and PMPU. For instance, You et al. [47] found that self-esteem was associated with mobile phone addiction through the mediating roles of social anxiety and interpersonal sensitivity. Li et al. [45] found that self-esteem was negatively associated with PMPU only for participants with high levels of interpersonal trust. The findings suggest that the association between self-esteem and PMPU is likely contingent upon specific psychological processes or conditional factors [45,47,48]. Consequently, to enhance our understanding of how self-esteem relates to PMPU, it is crucial to uncover the psychological mechanisms that form the basis of this association. 

The Interaction of Person-Affect-Cognition-Execution (I-PACE) model for addictive behaviors provides a comprehensive theoretical foundation for this study [49,50]. According to this theory, addictive behaviors can emerge and persist due to the interactions between predisposing variables, affective and cognitive responses, and coping styles. Specifically, the predisposing variables (e.g., self-esteem) function as susceptibility factors that contribute significantly to addictive behaviors. Affective responses to external or internal stimuli (e.g., FoMO) may be important mechanisms to explain the pathway through which predisposing variables are related to addictive behaviors. Moreover, several coping factors (e.g., rumination) may function as a moderating variable affecting the relationship between the predisposing variables and addictive behaviors [49]. Accordingly, we constructed a moderated mediation model to jointly examine the mediating role of FoMO and the moderating role of rumination in the association between self-esteem and PMPU. The findings would help illuminate how and under what conditions self-esteem links to PMPU, which contributes to a better understanding of the relationship between self-esteem and PMPU.

### 1.2. The Mediating Role of Fear of Missing Out

Fear of missing out (FoMO), referring to “a pervasive apprehension that others might be having rewarding experiences from which one is absent” [51] (p. 1841), may mediate the effect of self-esteem on PMPU. According to self-determination theory [52], people require relatedness as one of the fundamental psychological needs crucial for psychological well-being. People with high levels of FoMO generally suffer from relatedness, needs, frustration, and the accompanying negative emotional states (e.g., anxiety) [51]. They are reluctant to miss out on important information or novel experiences in their social network [51]. Thus, the unfulfilled need for relatedness may, in turn, urge them to keep up to date on others’ plans and activities by engaging with social media engagement [32,53]. Researchers have further elaborated on FoMO on social media, which describes the apprehension or preoccupation due to the inability to access online content or interact with others in a timely manner [54]. Mobile phones have already functioned as an effective tool for social media engagement. Therefore, high levels of FoMO could be associated with increases in mobile phone use. Empirical research has documented positive associations between FoMO and PMPU [32,55,56] and problematic social media use [53]. A meta-analytic study of 85 studies demonstrated that FoMO had a high and positive association with PMPU (r = 0.47) [57].

Moreover, FoMO is significantly related to self-esteem [55,58]. Specifically, people’s self-esteem levels are influenced by perceiving the extent to which they are valued by groups or social relationships [59]. People with low self-esteem generally perceive their relational value as low [37], which may result in high vigilance toward potential social threats [60]. Such vigilance may cause individuals to constantly worry about missing out on rewarding experiences from others. A longitudinal study further documented that those with low self-esteem were more likely to experience an increase in FoMO during a 6-month period [58].

The I-PACE model postulates that affective and cognitive processes are important pathways to account for how people’s core characteristics are connected to specific internet-use disorders [49,50]. According to this theory, individuals who hold the core trait of low self-esteem are more susceptible to negative emotional and cognitive experiences such as low self-worth, depression, anxiety [37], sensitivity to rejection [61], and fear of exclusion [37,38,58]. These negative emotional and cognitive processes can prompt individuals to engage in some behaviors to avoid and alleviate these negative experiences, such as using mobile phones, which possibly leads to PMPU. Consistent with this theory, empirical studies have validated that FoMO mediates the association between certain personality traits and internet-use disorders. For instance, studies have shown that FoMO mediated the relationship between self-concept clarity and problematic smartphone use [62] and between self-esteem and problematic social media use [63]. The present study will further investigate the mediating role of FoMO in the association between self-esteem and PMPU. Taken together, we proposed the following hypothesis:

**Hypothesis 1.**  *FoMO will mediate the association between self-esteem and PMPU.*

### 1.3. The Moderating Role of Rumination

Based on the I-PACE model [49,50], rumination as an avoidant coping strategy in response to negative experiences [64] may moderate the association between self-esteem and PMPU. Rumination refers to one’s passive and repetitive thinking about the symptoms of distress, the causes, and the potential consequences of distress [65]. The response styles theory postulates that rumination will prolong and exacerbate one’s negative affect and its consequences through enhancing negative thinking, interfering with adaptive problem-solving, and eroding social support [65,66]. In support of this view, previous research has documented the risk-enhancing role of rumination [33,67]. For example, Liu et al. [33] found that rumination accentuated the association between attachment anxiety and PMPU, with the effect being stronger for individuals with higher rumination. A longitudinal study showed that rumination worsened the effects of childhood emotional maltreatment on adolescents’ sleep problems and the effects of sleep problems on their NSSI behaviors six months later [67]. Therefore, according to the response styles theory, rumination may exacerbate the direct and mediated pathways from low self-esteem to PMPU.

Some studies further investigated the interaction effect of self-esteem and rumination on individual physical and mental health; however, the results have been inconsistent [68,69]. For instance, some researchers found that the interaction between self-esteem and rumination was negatively related to adolescent depression [68]. This indicates that the association between low self-esteem and depression was stronger under the conditions of high levels of rumination. In contrast, some researchers found that the interaction between self-esteem and rumination had a positive association with sleep problems in adolescents diagnosed with depression, such that the association between low self-esteem and depression became weaker under the conditions of high rumination [69]. The mixed findings may be attributed to the distinct characteristics of the samples. Another possible explanation is that these studies focused only on overall rumination and did not distinguish between the two subtypes of rumination. Recent studies have shown that rumination consists of two components, brooding and reflection, showing different effects on psychosocial adaptation [70,71].

#### 1.3.1. The Moderating Role of Brooding

Brooding refers to passively dwelling on the symptoms and consequences of negative affect and comparing one’s current situation with some unachieved standard in a negative way [64,71]. It is assumed that brooding represents a maladaptive coping style that is associated with increased risk for mental health problems such as depression [70], suicide ideation [72], and substance use disorders [73,74]. In addition, brooding has been consistently found to exacerbate the relationship between unfavorable factors and individual adaptation [75,76,77]. For instance, Cox et al. [77] found that brooding moderated the relationship between stress and depression, and the relationship became stronger for adolescents with a greater tendency toward brooding. Since low self-esteem is viewed as a risk factor for FoMO and PMPU, brooding may aggravate the undesirable effects of low self-esteem on FoMO and PMPU; that is, when people passively and repetitively dwell on the distressing experiences associated with low self-esteem, they may experience heightened FoMO and resort to mobile phone use for relief or reassurance, probably leading to PMPU. Moreover, brooding may exacerbate the effect of FoMO on PMPU, motivating people to indulge in mobile phones to relieve negative experiences. Therefore, we put forward the following hypothesis:

**Hypothesis 2.**  *Brooding will moderate the direct effect of self-esteem on PMPU and the mediating effect of FoMO on the association between self-esteem and PMPU in that these effects would be stronger for individuals with higher brooding.*

#### 1.3.2. The Moderating Role of Reflection

Reflection refers to a purposeful attempt to engage in cognitive problem-solving to address the causes and consequences of one’s distress [71]. In contrast with brooding, reflection represents a relatively more adaptive aspect of rumination [71]. Empirical research has found that reflection is associated with a decreased risk for depression [71] and substance use disorders [73]. However, whether reflection necessarily leads to adaptive outcomes remains to be investigated [70,72,74]. Research examining the moderating effect of reflection has also shown mixed findings. For instance, some studies identified that reflection enhanced the positive effect of active coping on depressive symptoms [78], while it did not intensify the effects of stress on depression [70,77] or suicide ideation [76]. Moreover, Kane [79] found that reflection had an antagonistic effect on the relationship between posttraumatic stress disorder symptoms and posttraumatic growth, reversing the relationship from positive to negative. The findings suggest the complicated nature of reflection. Under such conditions, we will investigate whether reflection moderates (intensified or buffered) the direct effect of self-esteem on PMPU and the indirect effect of self-esteem on PMPU via FoMO. The hypothesis is presented as follows:

**Hypothesis 3.**  *Reflection will moderate the direct effect of self-esteem on PMPU and the mediating effect of FoMO on the association between self-esteem and PMPU in that these effects would be stronger for individuals with higher (or lower) reflection.*

### 1.4. The Current Study

**Figure 1:**
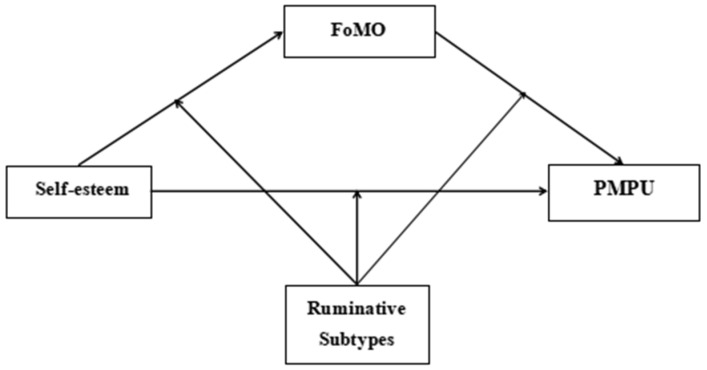
The proposed moderated mediation model. FoMO = fear of missing out, PMPU = problematic mobile phone use.

Building upon the I-PACE model and the response styles theory, the current study constructed a moderated mediation model (see Figure 1) to investigate the mechanisms underlying the association between self-esteem and PMPU. Specifically, this study aimed to address the following questions: (1) whether FoMO mediates the association between self-esteem and PMPU; and (2) whether the two subtypes of rumination (brooding, reflection) moderate the direct effect of self-esteem on PMPU and the mediating effect of FoMO.

## 2. Materials and Methods

### 2.1. Participants

We obtained approval from the Ethics Committee of Psychological Research of the authors’ university before the start of the survey. We used the convenience sampling method to select the target university in China and then used cluster random sampling to choose two to three majors in each grade (freshmen, sophomores, and juniors). Senior students were unavailable for the survey because they were busy with the postgraduate entrance exams or job hunting. Before data collection, we acquired informed consent from the participants and informed them that they had the option to consent or decline participation in the survey. They were also assured that their acquired information would remain confidential. Eight hundred and thirty-one undergraduate students participated in this paper-and-pencil survey in their classrooms. After removing invalid responses and incomplete data, 806 valid responses were obtained (completion rate = 96.99%). The sample size was in accordance with Cochran’s [80] formula for determining sample size, setting the acceptable sampling error at 0.05 and the confidence level of significance at 0.01 (z = 2.58). The mean age of the participants was 19.35 years (*SD* = 1.18, range = 17~23 years), with females making up 66.75% of the sample.

### 2.2. Measures

#### 2.2.1. Self-Esteem

Self-esteem was evaluated using the Rosenberg self-esteem scale [35]. The scale includes 10 items (e.g., “I take a positive attitude toward myself”) rated on a four-point scale, ranging from “1” = strongly disagree to “4” = strongly agree. Higher scores indicated higher levels of self-esteem. Due to potential ambiguity in understanding the eighth item within the Chinese cultural context, it was excluded from the analysis. The scale has been demonstrated to have satisfactory validity and reliability in Chinese adolescents and undergraduate students [81,82]. In our study, Cronbach’s alpha for this scale was 0.83.

#### 2.2.2. FoMO

FoMO was assessed using the 10-item scale developed by Przybylski et al. [51]. An example item includes “I fear others have more rewarding experiences than me.” Participants rated these items on a five-point scale (1 = not at all true of me, 5 = extremely true of me), with higher scores representing higher levels of a FoMO. This scale has shown good validity and reliability in Chinese adolescents and undergraduate students [83,84]. Cronbach’s alpha for this scale in the present study was 0.87.

#### 2.2.3. Rumination

Rumination was measured with the Ruminative Responses Scale [71]. The scale includes 10 items that assess the subscales of Brooding (5 items, e.g., thinking, “why do I have problems other people don’t have?”) and Reflection (5 items, e.g., “Analyze recent events to try to understand why you are depressed”), respectively. Each item is rated on a four-point scale (1 = never, 4 = always). Responses across the items on each of the subscales were calculated, and higher scores indicated greater levels of brooding/reflection. This scale has been demonstrated to be highly valid and reliable in Chinese undergraduate students and clinical samples [85,86]. Cronbach’s alpha for the total scale, the Brooding subscale, and the Reflection subscale in the current study were 0.82, 0.74, and 0.70, respectively.

#### 2.2.4. PMPU

The Mobile Phone Addiction Index (MPAI) [31] was administered to evaluate the levels of PMPU. The MPAI scale consists of 17 items that measure the four components of PMPU: “Inability to Control Craving” measures the extent to which individuals spend a significant amount of time on their mobile phones without being able to control it (loss of control, e.g., “You have attempted to spend less time on your mobile phone but are unable to”); “Feeling Anxious and Lost” measures the negative feelings that individuals experience when they reduce or stop mobile phone use (withdrawal, e.g., “You feel lost without your mobile phone”); “Withdrawal/Escape” measures using mobile phones to escape from real-life problems or to regulate their emotions (mood modification, e.g., “You have used your mobile phone to make yourself feel better when you were feeling down”); and “Productivity Loss” measures the impact of excessive mobile phone use on the efficiency of daily life and work (conflict, e.g., “You find yourself occupied on your mobile phone when you should be doing other things, and it causes problem”). These items were rated on a five-point scale, ranging from “1” = never to “5” = always. Higher scores represent greater levels of PMPU. This scale has exhibited good psychometric properties in Chinese adolescents and undergraduate students, indicated by high internal consistency and good concurrent validity [87,88,89]. The four-factor model has been demonstrated with a good model fit [88]: *χ*^2^/*df* = 6.44, *p* < 0.05, RMSEA = 0.03, SRMR = 0.004, CFI = 0.99, TLI = 0.99. In the current study, Cronbach’s alpha coefficient for the total scale and the four subscales was 0.87, 0.73, 077, 0.81, and 0.79, respectively, indicating satisfactory reliability.

### 2.3. Statistical Analysis

Firstly, we conducted data screening prior to statistical analysis and analyzed the 806 valid datasets for missing values. The results showed that the proportion of missing data cases for each survey item did not exceed 2.0%, and the proportion of missing data for each participant across all survey items did not exceed 8.5%. Further statistical analysis showed a total of 120 missing data points, accounting for 0.317% of the entire dataset (*N* = 37,882). According to some researchers, when dealing with missing data, the quality of the imputed values depends on factors such as the missing data mechanism, variable distribution, and correlations, in addition to the percentage of missingness [90]. Specifically, we used Little’s MCAR test to analyze the missing data mechanism. The results showed that the MCAR value reached a significant level: *χ*^2^ = 2882.93, *df* = 2.36, and *p* < 0.001, indicating that the missing mechanism was not Missing Completely at Random. Concerning variable distribution, the skewness and kurtosis values of the primary variables suggested they were close to a normal distribution, with the highest skewness value in absolute size being 0.61 and the highest kurtosis value in absolute size being 0.62. As for outliers, based on the criterion that the standard scores (Z-score) above 4 can be viewed as outliers in large samples (*N* > 80), no outliers were identified in our samples. Regarding the variable correlations, the results of this study (Table 1) showed significant correlations between self-esteem, FoMO, and PMPU. Except for the non-significant correlation between self-esteem and rumination, all other correlations between FoMO, PMPU, and rumination were significant. Based on the above research results, we found that the proportion of missing data was relatively low, the data missing mechanism in our study was not Missing Completely at Random, and the distribution of variables was approximately normal distribution with no outliers, which enhances the quality of missing data imputation to some extent. Therefore, we employed the Multiple Imputation method, which is more widely used and produces less biased estimates across different missing mechanisms [91].

Secondly, we performed descriptive analyses and Pearson correlation analyses using SPSS. 23.0 for preliminary analysis. Thirdly, we employed the SPSS macro PROCESS developed by Hayes [92] to examine the mediating effect of FoMO (Model 4) and the moderating effects of ruminative subtypes in the relationship between self-esteem and PMPU (Model 76). The PROCESS macro offered a comprehensive set of features for analyzing complex models involving both mediation and moderation effects and providing conditional indirect effects for moderated mediation models [92]. The bootstrapping approach with 5000 iterations further tested the significance of the mediation effect and the moderated mediation effects. These effects were considered significant if the 95% bias-corrected confidence intervals (95% CI) did not contain zero.

## 3. Results

### 3.1. Common Method Variance Test

Since the data were collected from participants’ self-reports on survey items, there may be potential common method bias. To address this, we implemented several procedural measures, such as selecting scales with established psychometric properties, incorporating reverse-scored items, employing varied rating scales, and conducting anonymous surveys [93]. We also performed Herman’s single-factor analysis to examine the possible common-method variance generated by the self-reported data collection method. Exploratory factor analysis was used to load all items into a single factor. The results showed that the single factor could explain about 17.81% of the variance, which was below the criteria value of 40%. Therefore, there was no significant common method variance in the present study.

### 3.2. Preliminary Analyses

As shown in Table 1, preliminary analyses included computing the means, standard deviations, and Pearson correlations for the main research variables. As expected, self-esteem was negatively associated with FoMO and PMPU. FoMO, overall rumination, two subtypes of rumination (brooding and reflection), and PMPU were positively intercorrelated. The correlations between self-esteem and rumination, as well as its two subtypes, were non-significant. As suggested by some researchers [94], a variable that exhibits weak correlations with the independent or dependent variable is less likely to be a mediating variable but has the potential to be a moderating variable. An ideal moderator variable should be independent, exhibiting insignificant or relatively small correlations with both the independent and dependent variables. Therefore, these correlation results suggest that these variables are suitable for further analyses of the mediation effect and moderated mediation effect. Moreover, given that gender and age were significantly associated with PMPU, they were included as covariables in the following analyses.

### 3.3. Testing for the Mediation Model

**Figure 2:**
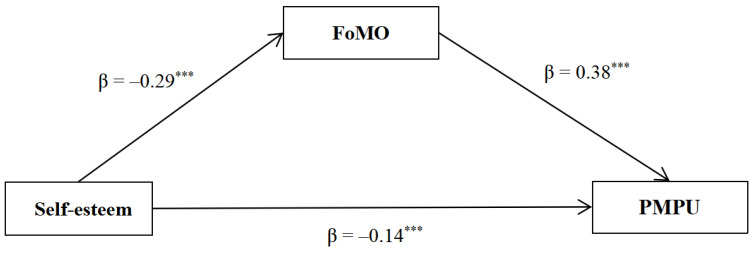
The mediating effect of FoMO in the association between self-esteem and PMPU. Standardized regression coefficients were displayed. *** *p* < 0.001.

The mediation effect analysis results (see Table 2 and Figure 2) revealed that after controlling for gender and age, self-esteem was negatively associated with PMPU and FoMO. When self-esteem and FoMO entered the prediction model of PMPU simultaneously, FoMO was positively associated with PMPU, and the negative association between self-esteem and PMPU was still significant. The bias-corrected Bootstrap approach further revealed that the indirect effect of self-esteem on PMPU through FoMO (indirect effect = −0.11, SE = 0.02, 95% CI = [−0.15, −0.07]) accounted for 44.28% of the total effect (total effect = −0.25, SE = 0.03, 95% CI = [−0.31, −0.18]. The results indicated that FoMO mediated the association between self-esteem and PMPU, which supported Hypothesis 1.

### 3.4. Testing for the Moderated Mediation Model

**Figure 3:**
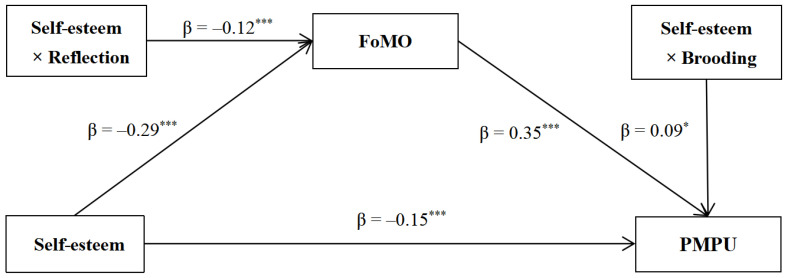
The moderating effects of rumination subtypes (brooding, reflection) in the association between self-esteem and PMPU. Standardized regression coefficients were displayed. * *p* < 0.05. *** *p* < 0.001.

The moderating roles of brooding and reflection were tested by performing a moderated mediation analysis in a whole model. The results (see Table 3 and Figure 3) revealed that after controlling for gender and age, self-esteem was negatively related to FoMO and PMPU, and FoMO was positively related to PMPU. In the prediction model of FoMO, the interaction between self-esteem and reflection was significantly related to FoMO (β = −0.12, SE = 0.04, *p* < 0.001), whereas the interaction between self-esteem and brooding did not significantly predict FoMO (β = 0.03, SE = 0.04, *p* = 0.43). In the prediction model of PMPU, the interaction between FoMO and brooding was significantly related to PMPU (β = 0.09, SE = 0.04, *p* < 0.05), whereas the interaction terms of self-esteem and brooding (β = 0.04, SE = 0.04, *p* = 0.34), self-esteem and reflection (β = −0.01, SE = 0.04, *p* = 0.68), and FoMO and reflection (β = −0.01, SE = 0.04, *p* = 0.72) were non-significant in predicting PMPU. 

**Figure 4:**
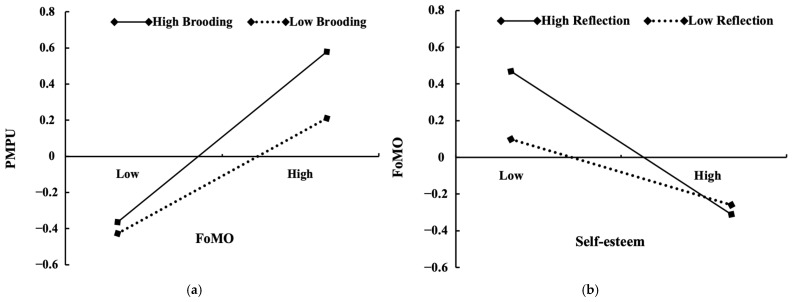
Testing the moderating roles of ruminative subtypes in the relation between self-esteem and PMPU: (**a**) Interaction between FoMO and brooding predicting PMPU at high (1 *SD* above the mean) and low levels of brooding (1 *SD* below the mean); (**b**) Interaction between self-esteem and reflection predicting FoMO at high (1 *SD* above the mean) and low levels of reflection (1 *SD* below the mean). Note that the y-axis values depict variations in the dependent variable resulting from interactions between the independent and moderating variables. The x-axis illustrates the “low” and “high” levels of the independent variable.

To depict the moderating effect of brooding, this study plotted the relationship between FoMO and PMPU at low (1 *SD* below the mean) and high levels of brooding (1 *SD* above the mean) (see Figure 4a). Simple slope tests showed that FoMO was positively related to PMPU for participants with low levels of brooding (β_simple_ = 0.29, *t* = 2.94, *p* < 0.01), and this relationship became much stronger for participants with higher levels of brooding (β_simple_ = 0.51, *t* = 7.06, *p* < 0.001). The conditional indirect effect tests further showed that at low (*M* − 1*SD*), medium (*M*), and high (*M* + 1*SD*) values of brooding, the conditional indirect effect of self-esteem on PMPU through FoMO was −0.08 [−0.14, −0.04], −0.10 [−0.14, −0.07], and −0.11 [−0.17, −0.06], respectively. Taken together, the results indicated that brooding moderated (strengthened) the indirect effect of self-esteem on PMPU via FoMO in that the indirect effect showed a stronger trend for participants with higher levels of brooding. Thus, Hypothesis 2 was partially supported. 

To depict the moderating effect of reflection, this study plotted the relationship between self-esteem and FoMO at low (1 *SD* below the mean) and high levels of reflection (1 *SD* above the mean) (see Figure 4b). Simple slope tests showed that self-esteem was negatively related to FoMO for participants with low levels of reflection (β_simple_ = −0.21, *t* = −2.82, *p* < 0.01), and this relationship became much stronger for participants with high levels of reflection (β_simple_ = −0.40, *t* = −5.17, *p* < 0.001). The conditional indirect effect tests further showed that at low (*M* − 1*SD*), medium (*M*), and high (*M* + 1*SD*) values of reflection, the conditional indirect effect of self-esteem on PMPU through FoMO was −0.06 [−0.11, −0.01], −0.10 [−0.14, −0.07], and −0.14 [−0.21, −0.08], respectively. Taken together, the results indicated that reflection moderated (strengthened) the indirect effect of self-esteem on PMPU via FoMO in that the indirect effect became stronger for participants with higher levels of reflection. Thus, Hypothesis 3 was partially supported.

## 4. Discussion

Based on the I-PACE model [49,50] and the response styles theory [65,66], the present study investigated FoMO and ruminative subtypes as possible mechanisms that link self-esteem to PMPU. The results showed that FoMO, acting as a mediator variable, accounted for “how” self-esteem was associated with PMPU and delineated a pathway of “Self-esteem→FoMO→PMPU”. Both subtypes of rumination, brooding and reflection, acting as moderator variables, explained under which conditions the mediating pathway “Self-esteem→FoMO→PMPU” became stronger (higher brooding or reflection) or weaker (lower brooding or reflection). These findings add to existing research by revealing in what ways and under what conditions self-esteem is associated with PMPU. 

### 4.1. The Mediation Effect of FoMO

Consistent with the excessive reassurance pathway leading to PMPU [7] and prior studies [46,47], the present research validates the prominent role of low self-esteem in the development of PMPU. That is, individuals who have negative perceptions and evaluations of themselves have the need to seek reassurance and establish interpersonal connections, which drives them to develop dependence on their mobile phones and even develop PMPU. This study also revealed that self-esteem was negatively associated with FoMO, which in turn was positively associated with PMPU. That is, FoMO functioned as a mediator that accounted for the pathway through which self-esteem was associated with PMPU, which supported Hypothesis 1. The results confirmed the I-PACE model [49,50] and prior studies that FoMO as affective responses may be an important pathway through which self-related core traits (predisposing variables) influence internet-use disorders [62,95]. 

Specifically, the results showed that FoMO was associated with high levels of PMPU, which is congruent with prior studies [55,96]. According to the compensatory internet use theory, people are motivated to engage in internet activities to compensate for their psychosocial problems [97]. Thus, individuals with a high FoMO tend to develop mobile phone overuse to fulfill their needs for relatedness and connection to social networks [32,53]. Moreover, self-esteem was found to be negatively associated with FoMO, which aligns with prior studies [63,98]. The results can be accounted for by terror management theory [99,100]. The theory posits that people have a central motive to live up to cultural values and identify with groups [37]. Such identification is associated with high self-esteem, which in turn buffers against deep-death anxiety [37]. Low self-esteem, by contrast, may be associated with increased anxiety elicited by the factors that signify human mortality, such as destruction, failure, exclusion, or abandonment [101,102,103]. FoMO can be viewed as a subtype of anxiety, referring to a disseminated apprehension about missing rewarding experiences [51]. Therefore, low self-esteem can act as a diathesis for FoMO. 

### 4.2. The Moderation Effects of Ruminative Subtypes

The present study demonstrated the moderating roles of ruminative subtypes in the indirect effect of self-esteem on PMPU through FoMO, which partially supported Hypothesis 2 and 3. The results showed that both brooding and reflection, two subtypes of rumination, intensified the indirect relationship between self-esteem and PMPU (Self-esteem→FoMO→PMPU). The indirect effect became stronger for individuals with higher levels of either brooding or reflection, which confirmed the risk-enhancing effect of rumination [67]. This finding aligns with both the I-PACE model and the Response Styles Theory [66], suggesting that rumination, as a coping mechanism, can amplify the impact of predisposing factors on individuals’ affective responses and behavioral adaptation. However, there were differences regarding the specific moderating effects of the two ruminative subtypes. Specifically, brooding was found to exacerbate the association between FoMO and PMPU (the second stage of the mediation process), while reflection was found to exacerbate the association between self-esteem and FoMO (the first stage of the mediation process). The findings suggest that the two subtypes of rumination are closely connected yet may exert different effects on individuals’ psychological and behavioral adaptation [70,71]. Thus, examining the two subtypes of rumination rather than overall rumination can help illuminate how specific ruminative types influence the relationship between self-esteem and PMPU.

First, the results showed that brooding intensified the association between FoMO and PMPU. This association became stronger at higher levels of brooding compared with lower levels of brooding. The results lend support to prior studies demonstrating that brooding exacerbates the impacts of negative experiences on individual psychosocial adaptation [75,76,77]. In the present study, individuals with high levels of FoMO generally worry and follow other people’s activities and social information as a method of compensation. The high tendency of brooding may urge them to passively contemplate these adverse experiences and dwell on thoughts of self-blame [71], leading them to be overly vigilant toward the possible missing information from others. The heightened FoMO may, in turn, drive individuals to overly rely on mobile phones to evade or relieve their negative experiences. The findings indicated that brooding functioned as a maladaptive coping mechanism that aggravated the effect of FoMO on PMPU. However, brooding did not intensify or weaken the relationship between self-esteem and FoMO, such that the effect of self-esteem on FoMO was independent of individual brooding. The results suggest that brooding may be more effective in stimulating the effects of emotional experiences while having a limited effect on self-related core traits.

Second, reflection was found to be a catalyst in the relationship between self-esteem and FoMO. Compared with low levels of reflection, the relationship became stronger under the conditions of higher levels of reflection. Prior studies have suggested that reflection involves the intentional engagement of problem-solving [71]. However, the adaptive role of reflection may vary depending on the context in which this coping style occurs [78,104]. For instance, Marroquín et al. [78] found that for individuals who actively coped with stressors, their high tendency to reflect upon problems played an adaptive and beneficial role, whereas, among those low in active coping, the high levels of reflection were associated with increased depressive symptoms. Based on this inference, the moderating effect of reflection in the current study can be interpreted in two ways. On the one hand, the results showed that reflection enhanced the beneficial effect of high self-esteem on FoMO. Individuals who exhibit both high levels of self-esteem and reflection are inclined to actively monitor their feelings and cognitively evaluate their relatedness needs in an attempt to address their problems. Such a tendency protects people from developing negative experiences such as FoMO. On the other hand, the results indicate that high levels of reflection aggravated the detrimental effect of low self-esteem on FoMO, with the highest levels of FoMO being observed in those with low levels of self-esteem and high levels of reflection simultaneously. Low self-esteem, as a negative self-evaluation, has been established as a prominent factor in individual emotional problems [37]. Thus, for individuals who hold negative self-views (i.e., low self-esteem), even though they purposefully engage in cognitive problem-solving driven by high reflection [71], such attempts seem to be unsuccessful in generating solutions [105]. The failed attempts may exacerbate people’s negative self-appraisal, resulting in subsequent unfavorable experiences. The result is consistent with prior studies showing that reflection increases the risk of depression when combined with high levels of brooding and stress [104]. Thus, compared with individuals with low reflection, those with high reflection may be more susceptible to relatedness need frustration and FoMO when they present with low self-esteem. Moreover, the findings showed that reflection could not moderate the effect of FoMO on PMPU, indicating that the effect of FoMO on PMPU was independent of the levels of reflection. The results suggest that reflection, which involves cognitive engagement, may be more effective in stimulating the effects of self-related core traits than emotional experiences.

Moreover, the findings showed that neither brooding nor reflection moderated (intensified or weakened) the direct effect of self-esteem on PMPU. Such unexpected findings may be attributed to the dominant effect of self-esteem and the less potent effect of rumination on PMPU. That is, self-esteem has been such a potent predictor of PMPU that people’s tendency to dwell on the symptoms and consequences of their problems did not vary their levels of PMPU. Moreover, it is possible that rumination as a response style is more closely associated with the development of emotional problems [70,72] compared with behavioral problems [73]. Therefore, although brooding and reflection did not impact the direct effect of self-esteem on PMPU, they could moderate the indirect effect of self-esteem on PMPU through FoMO.

### 4.3. Theoretical and Practical Implications

The present study constructed a moderated mediation model and verified the independent and joint effects of predisposing factors (self-esteem), affective responses (FoMO), and coping factors (rumination) on PMPU. The findings support the I-PACE model and response style theory and facilitate the application of these theoretical models to technology-mediated behaviors. Moreover, the findings extend previous research by uncovering the psychological processes underlying the association between self-esteem and PMPU. Specifically, our findings reveal the pathway through which self-esteem was related to PMPU and under what conditions this relationship between self-esteem and PMPU became stronger or weaker. Since few studies, to date, have focused on the moderating roles of ruminative subtypes in the relationship between self-esteem and PMPU, this study contributes to existing research by investigating the distinct roles of brooding and reflection, specifically. Regarding the methodological implications, the findings highlight the importance of constructing a conceptual model based on existing theories and employing rigorous research design when investigating issues such as PMPU.

In addition, this study offers important practical implications for the prevention and intervention of PMPU. First, the study contributes to the development and implementation of both universal and selective preventive intervention. Unlike previous intervention programs that target specific groups of diagnosed individuals, our research highlights the importance of broadening the focus to include the susceptible groups with certain psychological characteristics. For instance, more attention should be given to susceptible groups with low self-esteem, high FoMO, and especially those who exhibit both low self-esteem and high reflection or high FoMO and high brooding. Second, the present study provides suggestions for the development of targeted intervention strategies to address these psychological factors associated with PMPU. 

Specifically, enhancing self-esteem can be beneficial in preventing and intervening in PMPU due to its predisposing role in PMPU. Prior research has indicated that CBT can improve self-esteem by integrating cognitive and behavioral skills, modifying negative self-perceptions and beliefs, and reshaping positive self-beliefs [106]. Moreover, addressing FoMO can be an effective strategy for reducing PMPU, given its mediating effect between self-esteem and PMPU. Researchers have developed a range of methods and intervention techniques to help alleviate FoMO levels [54]. In addition, this study confirms the moderating roles of the two components of rumination in the “Self-esteem→FoMO→PMPU” pathway. This implies that regulating rumination levels is crucial, especially for individuals with low self-esteem and high reflection, as well as those with high FoMO and high brooding. Prior research has shown that psychological interventions, such as rumination-focused CBT, metacognitive therapy, and mindfulness-based CBT, can effectively reduce rumination levels by regulating individual cognition, attitudes, and behaviors [107].

### 4.4. Limitations and Future Research

Several limitations need to be noted. First, the cross-sectional design of the present study could not reveal the causal relationships. More longitudinal designs and experimental methods are needed in future research to ascertain the relationships between the core variables. Second, we tested our research model on Chinese university students by convenience sampling, which may limit the generalizability of our findings [2]. The findings of our research need to be validated in other populations, such as those with different cultural contexts and age groups. Moreover, we did not assess the participants’ prior psychopathological conditions during recruitment. Future research should collect psychopathological data from participants and further examine the moderated mediation model constructed in this study within clinical samples. Third, the research data was gathered through self-report questionnaires, which may be susceptible to biases such as social desirability bias. To enhance the robustness of findings in future studies, it would be beneficial to incorporate data from multiple sources (e.g., teachers, parents, and friends) and integrate objective mobile phone use data, such as automatically logged behavior records [108,109]. Fourth, prior research has established the different associations between implicit self-esteem (measured by the Implicit Association Test) and explicit self-esteem (measured by the Rosenberg Self-Esteem Scale) with PMPU [44]. Therefore, it is essential for future studies to integrate explicit self-reports with the Implicit Association Test to examine the relationship more comprehensively between participants’ implicit and explicit self-esteem and PMPU.

## 5. Conclusions

Based on the I-PACE model and the response styles theory, the present study built a moderated mediation model to investigate the mechanisms underlying the relationship between self-esteem and PMPU. Results showed that lower self-esteem was associated with higher levels of PMPU. FoMO mediated the relationship between self-esteem and PMPU. Moreover, brooding moderated the relationship between FoMO and PMPU, and reflection moderated the relationship between self-esteem and FoMO. Both brooding and reflection aggravated the mediating effect of FoMO on the relationship between self-esteem and PMPU.

## Figures and Tables

**Table 1:** Descriptive statistics and correlations between variables.

Variables	1	2	3	4	5	6	7	8
1. Gender	—							
2. Age	−0.01	—						
3. Self-esteem	0.05	0.02	—					
4. FoMO	−0.08 *	−0.12 ***	−0.30 ***	—				
5. PMPU	0.10 **	−0.10 **	−0.24 ***	0.41 ***	—			
6. Rumination	0.01	−0.02	0.02	0.23 ***	0.14 ***	—		
7. Brooding	0.04	−0.04	0.06	0.22 ***	0.16 ***	0.90 ***	—	
8. Reflection	−0.02	-0.002	−0.03	0.20 ***	0.09 *	0.91 ***	0.64 ***	—
*M*	0.67	19.35	2.83	2.14	2.55	2.32	2.45	2.20
*SD*	0.47	1.18	0.46	0.70	0.64	0.49	0.53	0.56

Note. *N* = 806. FoMO = fear of missing out, PMPU = problematic mobile phone use. Gender was dummy coded such that 0 = male and 1 = female. * *p* < 0.05, ** *p* < 0.01, *** *p* < 0.001.

**Table 2:** Testing FoMO as a mediator in the relation between self-esteem and PMPU.

Predictors	Model 1 (PMPU)			Model 2 (FoMO)			Model 3 (PMPU)		
	β	95% CI	*t*	β	95% CI	*t*	β	95% CI	*t*
Gender	0.23	[0.09, 0.37]	3.21 **	−0.15	[−0.29, −0.01]	−2.15 *	0.29	[0.16, 0.42]	4.31 ***
Age	−0.08	[−0.14, −0.02]	−2.77 **	−0.10	[−0.16, −0.04]	−3.55 ***	−0.04	[−0.09, 0.01]	−1.55
Self-esteem	−0.25	[−0.31, −0.18]	−7.29 ***	−0.29	[−0.36, −0.22]	−8.68 ***	−0.14	[−0.20, −0.07]	−4.18 ***
FoMO							0.38	[0.31, 0.44]	11.35 ***
*R* ^2^	0.08			0.11			0.21		
*F*	23.38 ***			31.84 ***			52.52 ***		

Note. *N* = 806. Standardized regression coefficients were reported. Bootstrap sample size = 5000. * *p* < 0.05. ** *p* < 0.01. *** *p* < 0.001.

**Table 3:** Testing FoMO as a mediator and brooding and reflection as moderators in the relation between self-esteem and PMPU.

Predictors	Model 1 (FoMO)			Model 2 (PMPU)		
	β	95% CI	*t*	β	95% CI	*t*
Gender	−0.17	[−0.30, −0.03]	−2.41 *	0.28	[0.14, 0.41]	4.13 ***
Age	−0.09	[−0.15, −0.04]	−3.37 ***	−0.04	[−0.09, 0.01]	−1.56
Self-esteem	−0.29	[−0.35, −0.22]	−8.75 ***	−0.15	[−0.22, −0.09]	−4.65 ***
Brooding	0.20	[0.11, 0.28]	4.63 ***	0.13	[0.05, 0.21]	3.15 **
Reflection	0.08	[−0.005, 0.16]	1.85	−0.06	[−0.14, 0.02]	−1.39
Self-esteem × Brooding	0.03	[−0.04, 0.10]	0.79	0.04	[−0.04, 0.11]	0.96
Self-esteem × Reflection	−0.12	[−0.19, −0.05]	−3.42 ***	−0.01	[−0.08, 0.06]	−0.41
FoMO				0.35	[0.28, 0.42]	10.00 ***
FoMO × Brooding				0.09	[0.01, 0.17]	2.29 *
FoMO × Reflection				−0.01	[−0.09, 0.06]	−0.36
*R* ^2^	0.18			0.22		
*F*	24.36 ***			23.06 ***		

Note. * *p* < 0.05. ** *p* < 0.01. *** *p* < 0.001.

## Data Availability

The data that support the findings of this study are available from the corresponding author upon reasonable request.
